# Linking Xylem Hydraulic Conductivity and Vulnerability to the Leaf Economics Spectrum—A Cross-Species Study of 39 Evergreen and Deciduous Broadleaved Subtropical Tree Species

**DOI:** 10.1371/journal.pone.0109211

**Published:** 2014-11-25

**Authors:** Wenzel Kröber, Shouren Zhang, Merten Ehmig, Helge Bruelheide

**Affiliations:** 1 Institute of Biology, Geobotany and Botanical Garden, Martin-Luther-University Halle-Wittenberg, Halle (Saale), Germany; 2 State Key Laboratory of Vegetation and Environmental Change, Institute of Botany, the Chinese Academy of Sciences, Beijing, China; 3 German Centre for Integrative Biodiversity Research (iDiv) Halle-Jena-Leipzig, Leipzig, Germany; Wesleyan University, United States of America

## Abstract

While the fundamental trade-off in leaf traits related to carbon capture as described by the leaf economics spectrum is well-established among plant species, the relationship of the leaf economics spectrum to stem hydraulics is much less known. Since carbon capture and transpiration are coupled, a close connection between leaf traits and stem hydraulics should be expected. We thus asked whether xylem traits that describe drought tolerance and vulnerability to cavitation are linked to particular leaf traits. We assessed xylem vulnerability, using the pressure sleeve technique, and anatomical xylem characteristics in 39 subtropical tree species grown under common garden conditions in the BEF-China experiment and tested for correlations with traits related to the leaf economics spectrum as well as to stomatal control, including maximum stomatal conductance, vapor pressure deficit at maximum stomatal conductance and vapor pressure deficit at which stomatal conductance is down-regulated. Our results revealed that specific xylem hydraulic conductivity and cavitation resistance were closely linked to traits represented in the leaf economic spectrum, in particular to leaf nitrogen concentration, as well as to log leaf area and leaf carbon to nitrogen ratio but not to any parameter of stomatal conductance. The study highlights the potential use of well-known leaf traits from the leaf economics spectrum to predict plant species' drought resistance.

## Introduction

The worldwide leaf economics spectrum (LES) represents an important framework of trade-offs between key functional leaf traits [1]. It describes different strategies of carbon capture among vascular land plants, from that of short-lived leaves with high photosynthetic capacity per leaf mass, to long-lived leaves with low mass-based carbon assimilation rates. Mass-based photosynthetic capacity is positively related to mass-based leaf nitrogen concentration (LNC) and to specific leaf area [2] and is negatively related to leaf life span [1]. Many studies have confirmed the global validity of these trade-off patterns [3]–[5], and Osnas *et al.* [6] recently demonstrated that such relationships between traits in the LES result from relationships to leaf area and from normalizing area-proportional traits by leaf mass. Kröber & Bruelheide [7] have demonstrated that there are additional dimensions to plants' functional traits that are orthogonal to the LES. They found parameters of stomatal regulation (derived from stomatal conductance - vapor pressure deficit relationships), stomatal density and stomatal size to be independent from the LES.

So far, reported relationships between traits of the LES and those of plant organs other than leaves, such as wood or roots, are equivocal. For example, Baraloto *et al.* [8] found the main axes in leaf and wood traits to be decoupled, while Freschet *et al.* [9] provided evidence for a tight relationship between the main dimensions of the leaf, stem and root economics spectra. However, from a 'whole plant' perspective, tight relationships would be expected for those leaf and wood traits that determine a plant's tolerated minimum water potential, because the plant's water status links a multitude of physiological processes [10]. Choat *et al.* [11] and Poorter *et al.* [12] reported that leaves with high specific leaf area (SLA) were linked to stems with low wood density. Similar to wood density, hydraulic xylem properties would also be expected to be correlated across roots, stems and leaves. In particular, a high photosynthetic capacity of leaves, as expressed by high SLA, should be associated with high xylem hydraulic conductivity to facilitate sufficient water supply required for high stomatal conductance. Accordingly, in a study on ten tropical tree species in Panama, Sack and Frole [13] reported that leaf hydraulic resistance was strongly linked to leaf venation and mesophyll structure. The relationship between hydraulic conductivity and leaf venation was also confirmed across 43 species worldwide [14]. However, Sack *et al*. [15] argued that leaf hydraulic conductance might be mechanistically independent from the LES, but might be linked statistically as both hydraulic conductance and LES traits affect mass-based photosynthesis.

Besides being hydraulically efficient, another required feature of the vascular plumbing network is drought resistance. In this regard, species with stress-resistant leaves, as indicated by low SLA values, should be expected to have stress-resistant wood. Wood stress resistance is reflected in high wood density, which is thought to confer a higher tolerance from shade, wind, herbivores and drought [16]. In particular, drought resistance determined by measuring xylem vulnerability to cavitation should be correlated between leaves and wood, because cavitation is a persistent hazard under drought stress and affects leaves and wood [17]. Sustaining low water potentials requires high cavitation resistance of conduits, as derived from xylem vulnerability curves [18]. These curves allow quantifying the specific xylem hydraulic conductivity of the xylem (K_S_) and the xylem pressure at which 50% loss of the maximum specific xylem hydraulic conductivity occurs (Ψ_50_). Ψ_50_ is mainly determined by pit size and structure [19],[20]. However, low water potentials are transmitted throughout the whole plant, from the point where the water-pathway ends and the regulation of the water flow takes place to the xylem, where cavitation occurs. Thus, Ψ_50_ should be reflected in functional leaf traits and parameters of stomatal regulation.

Tree species that are able to endure severe drought periods have characteristic leaves. The leaves are tough and have a high leaf dry matter content (LDMC), allowing them to sustain low water potentials [21],[22]. Such species should likewise be characterized by low Ψ_50_ values. SLA is inversely related to LDMC, in that it decreases with drought resistance [23],[24], and it would be expected to scale negatively with Ψ_50_. In addition, cavitation vulnerability should also be related to stomatal regulation, because cavitation-sensitive and -insensitive species would be expected to close their stomata at low and high vapor pressure deficits, respectively [25]–[27]. Such parameters of stomatal closure have recently been provided by Kröber & Bruelheide [7] for the same 39 species used also in the current study. The authors measured daily courses of stomatal conductance (g_s_) with porometry in the same plots as in the present study, and modeled the species-specific g_s_ ∼ vapor pressure deficit (VPD) relationships. They found that mean g_s_ can be predicted from leaf traits that reflect the LES, with a positive relationship to LNC and a negative relationship to leaf carbon to nitrogen ratio. In contrast, the maximum of the g_s_ ∼ VPD curve was unrelated to traits of the LES and increased with leaf carbon concentration (LCC) and vein length. The VPD at which g_s_ was down-regulated, characterized by the point of inflection of the g_s_ ∼ VPD curve at high VPD, was lower for species with higher stomatal density and lower leaf carbon concentration. In addition to leaf trait measurements, we use these parameters of stomatal control from Kröber & Bruelheide [7] to predict xylem hydraulics.

The objective of our study was to quantify hydraulic conductivity and Ψ_50_ from xylem vulnerability curves, making use of the common garden situation of the BEF-China experiment. Comparing 39 broad-leaved tree species, we hypothesized that (1) leaf traits describing the leaf economics spectrum are related to specific xylem hydraulic conductivity and cavitation resistance. Accordingly, we expected that (2) evergreen species characterized by low SLA and high LDMC are more resistant to cavitation, i.e. have lower Ψ_50_ values than deciduous species, Finally, (3) we tested the hypothesis that parameters of stomatal regulation, such as maximum stomatal conductance, the vapor pressure deficit (VPD) at maximum stomatal conductance and VPD at which stomatal conductance is down-regulated, are related to high xylem hydraulic conductivity.

## Materials and Methods

### Study Site

**Figure 1:**
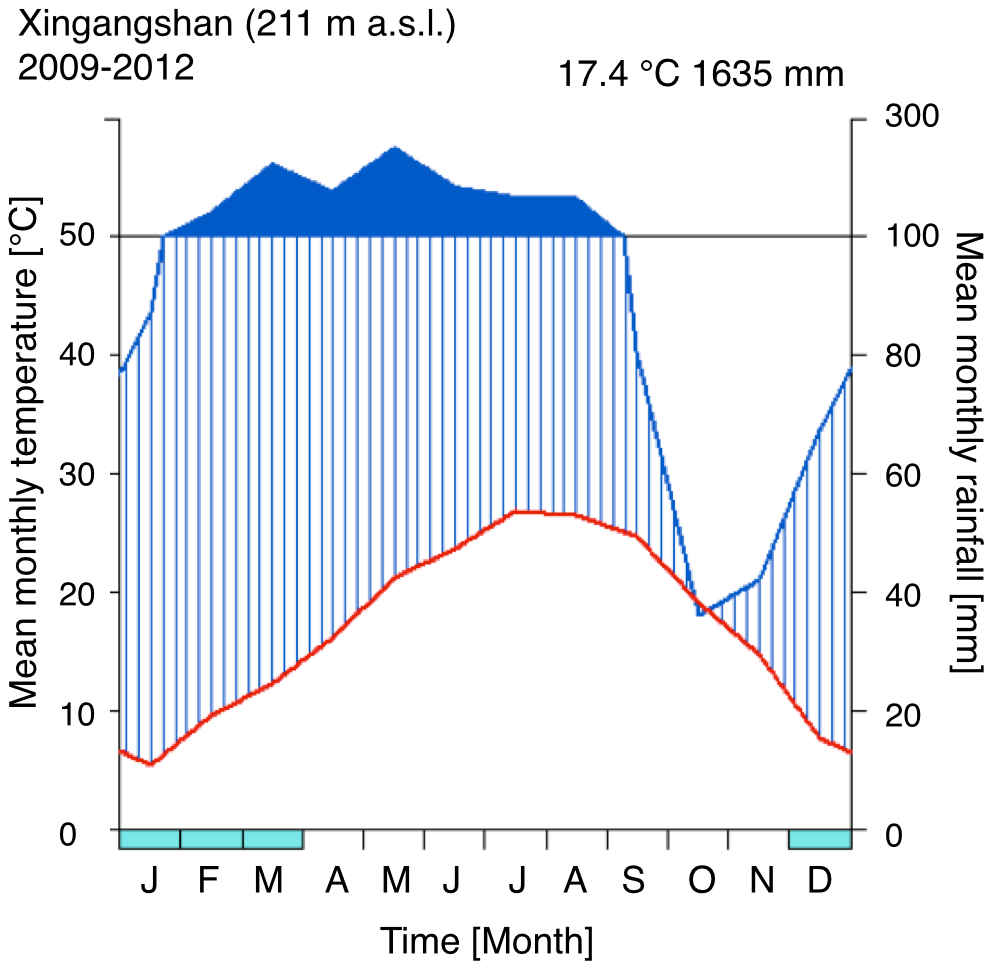
Climate diagram according to Walter & Lieth [28] of Xingangshan, the location of the experimental sites. Elevation: 211 m above sea level. Observation period was March 2009 to October 2012. Mean annual temperature was 17.4°C and total annual precipitation was 1635 mm. Monthly precipitation below 100 mm is scaled 2∶1 with mean monthly temperature (vertically hatched) and above 100 mm 15∶1. Turquoise bars below the x-axis show the months where frosts can occur (when absolute monthly minimums are equal or lower than 0°C). Climate data were recorded by a meteorological station established at the very center of the experimental site (Kühn, unpublished).

The study was conducted in the BEF-China project, which is a biodiversity-ecosystem functioning experiment based in Jiangxi Province, southeast China (http://www.bef-china.de; 29.08–29.11 N, 117.90–117.93 E). The climate at the experimental site is subtropical with moderately cold and dry winters and warm summers. Based on data of meteorological stations established at the sites, mean annual temperature was 17.4°C and mean annual precipitation was 1635 mm (Fig. 1). Across an area of 38 ha, 219,000 trees were planted at different levels of species richness [29]. The diversity gradient spans from monoculture to two, four, eight, 16 and up to 24 species per plot. The 39 tree species included in the study (see Table 1) are representative of the local natural broadleaved subtropical forest community [30],[31], and the trees assessed had already reached an age of four or five years at the time when our study was carried out. Using young even-aged trees in a common garden situation allowed for controlling for confounding factors, such as different ontological stage, but also allowed to sample leaves and branches at a standardized height above ground. No specific permissions were required for these locations and activities. The field studies did involve neither endangered nor protected species.

**Table 1:** Tree species planted in the BEF-China experiment and included in this study.

Species name	Family	Abbreviation	Leaf habit
*Acer davidii* Franch.	Aceraceae	Ad	d
*Ailanthus altissima* (Miller) Swingle	Simaroubaceae	Aa	d
*Alniphyllum fortunei* (Hemsl.) Makino	Styracaceae	Af	d
*Betula luminifera* Winkl.	Betulaceae	Bl	d
*Castanopsis eyrei* (Champion ex Bentham) Tutcher	Fagaceae	Ce	e
*Castanopsis fargesii* Franch.	Fagaceae	Cf	e
*Castanea henryi* (Skan) Rehd. et Wils.	Fagaceae	Ch	d
*Castanopsis sclerophylla* (Lindley & Paxton) Schottky	Fagaceae	Cs	e
*Celtis biondii* Pamp.	Cannabaceae	Cb	d
*Choerospondias axillaris* (Roxb.) Burtt et Hill	Anacardiaceae	Ca	d
*Cinnamomum camphora* (Linn.) Presl	Lauraceae	Cc	e
*Cyclobalanopsis glauca* (Thunberg) Oersted	Fagaceae	Cg	e
*Cyclobalanopsis myrsinifolia* (Blume) Oersted	Fagaceae	Cm	e
*Daphniphyllum oldhamii* (Hemsl.) Rosenthal	Daphniphyllaceae	Do	e
*Diospyros japonica* Siebold & Zuccarini	Ebenaceae	Dj	d
*Elaeocarpus chinensis* (Gardn. et Chanp.) Hook. f. ex Benth.	Elaeocarpaceae	Ec	e
*Elaeocarpus glabripetalus* Merr.	Elaeocarpaceae	Eg	e
*Elaeocarpus japonicus* Sieb. et Zucc.	Elaeocarpaceae	Ej	e
*Idesia polycarpa* Maxim.	Flacourtiaceae	Ip	d
*Koelreuteria bipinnata* Franch.	Sapindaceae	Kb	d
*Liquidambar formosana* Hance	Altingiaceae	Lf	d
*Lithocarpus glaber* (Thunb.) Nakai	Fagaceae	Lg	e
*Machilus grijsii* Hance	Lauraceae	Mg	e
*Machilus leptophylla Hand.-Mazz.*	Lauraceae	Ml	e
*Machilus thunbergii Sieb. et Zucc.*	Lauraceae	Mt	e
*Manglietia fordiana* (Oliver) HuY.W.Law	Magnoliaceae	Manf	e
*Melia azedarach* Linn.	Meliaceae	Ma	d
*Meliosma flexuosa* Blume	Sabiaceae	Mf	d
*Nyssa sinensis* Oliver	Nyssaceae	Ns	d
*Phoebe bournei* (Hemsl.) Yen C. Yang,	Lauraceae	Pb	e
*Quercus acutissima* Carruthers	Fagaceae	Qa	d
*Quercus fabri* Hance	Fagaceae	Qf	d
*Quercus phillyreoides* A. Gray	Fagaceae	Qp	e
*Quercus serrata* Murray	Fagaceae	Qs	d
*Rhus chinensis* Mill.	Anacardiaceae	Rc	d
*Sapindus saponaria* Linn.	Sapindaceae	Sd	d
*Triadica cochinchinensis* Loureiro	Euphorbiaceae	Tc	d
*Triadica sebifera* (L.) Small	Euphorbiaceae	Ts	d
*Schima superba* Gardn. et Champ.	Theaceae	Schs	e

Species names are in accordance with nomenclature in The Flora of China (http://flora.huh.harvard.edu/china). d =  deciduous, e =  evergreen.

### Tree Species and Vulnerability Curves

**Figure 2:**
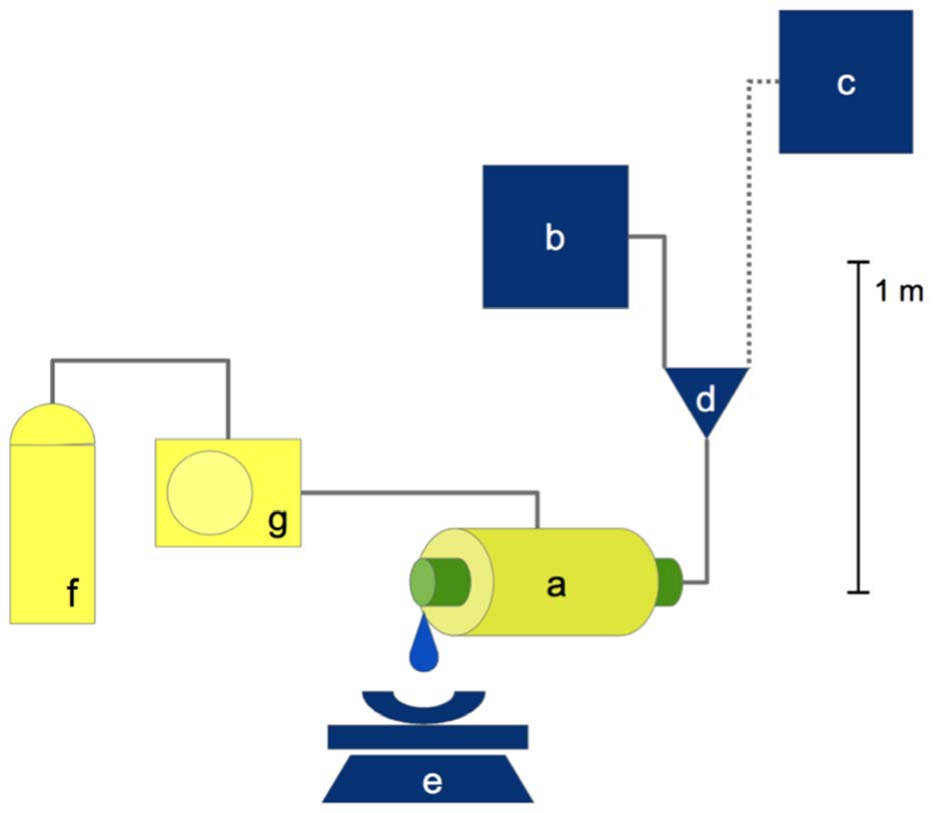
Simplified overview of the xylem hydraulics measurement installation. A) Pressure chamber with the stem segment, B) tank with the perfusion solution, C) flush tank, D) three-way stopcock, E) electronic scale, F) nitrogen pressure cylinder, and G) pressure gauge.

We randomly chose three individuals per species in the high-diversity plots, with one individual per species being sampled per plot. This enabled us to minimize time between sample cutting and lab procession of samples because different species grew in close proximity to each other. Xylem conductivity and vulnerability and leaf stomatal conductance (see below) were measured on the same plots, but not explicitly on the same individuals. The sampling and measurements on xylem hydraulics were conducted in August - October 2012. This period was characterized by monthly mean temperatures of about 20°C and a monthly precipitation of 40 mm (Fig. 1), which involved dry spells of several weeks, typically resulting in midday depressions of stomatal conductance. Samples were always taken in the early morning hours between 6 and 8 am, when relative humidity was still high (70–95% Rh) and temperatures were around 20°C. Measurements of leaf water potentials were made in spring 2012, using a PMS M1000 Scholander pressure chamber. These data showed that water potentials were well above −2 MPa, and for many species>−1 MPa. A twig with no leaves, buds or branches, around 15 cm in length and 5–15 mm diameter was cut and immediately immersed in water. We are aware that maximum vessel length of some of the species might be larger than 15 cm [32], which would result in overestimating specific xylem hydraulic conductivity (K_S_) and the absolute value of the xylem pressure at which 50% loss of the maximum specific xylem hydraulic conductivity occurs (Ψ_50_). However, it has been shown that extreme vessel lengths are very rare [33]. In any case, obtaining non-ramified twigs longer than 15 cm would have been impossible in most species. After transportation to the lab, the stem pieces were then placed into a double-ended pressure sleeve (PMS M1000 Scholander pressure chamber) in the laboratory following established protocols [18],[34] (Fig. 2). Xylem vulnerability was measured within at maximum four hours after cutting. Increasing the air pressure in the cavitation chamber was used to simulate increasingly negative xylem sap pressures [35]. Before the measurements were taken, each twig segment was treated for one hour with perfusion solution pressurized at 0.15 MPa in order to flush out air from older embolism events and any potential air entry into the xylem during the cutting and handling of samples. We used 10 mM citric acid perfusion solution, using filtered and demineralized water to prevent any blockages caused by microorganisms.

Specific xylem hydraulic conductivity (K_S_) was measured as the mass of flow-through perfusion solution through the piece of wood per unit of time and per cross-sectional area of the twig. The measurements were started at ambient air pressure and repeated as pressure was increased in increments of 0.5 MPa up to 7 MPa, or to the pressure at which no more perfusion solution flow was encountered.

### Measurements of Stomatal Conductance

Data on stomatal conductance were taken from [7]. For the measurements of stomatal conductance the authors had randomly selected twelve high-diversity plots (with 16 or 24 species). Eleven to 23 individuals of all species were measured per plot. In total, 3,290 measurements of stomatal conductance were made in August to October 2010, May/June 2011 and August to October 2011. Each of the 39 species was represented between at least three and at maximum 13 individuals, resulting in 218 individuals in total. This non-balanced sampling design resulted from measuring daily course of stomatal conductance, which required walking time to be minimal. The repeated measurements were taken on the same leaf, which was fully developed, undamaged and fully exposed to the sun. Daily courses of stomatal conductance were produced for all species in every plot. Stomatal conductance was recorded with an SC1 porometer (Decagon). Air temperature and relative humidity was measured simultaneously using a T2 thermo-hygrometer (Trotec). Vapor pressure deficit (VPD) was calculated following the August-Roche Magnus formula. The daily courses of all different individuals from all different daily courses were then aggregated to one g_s_ ∼ VPD relationship which included all data for one species. Mean and maximum stomatal conductance (g_smax_) could then be estimated per species. The species-specific g_s_ ∼ VPD relationships were modeled by regressing the logits of g_s_/g_smax_ to VPD and the quadratic term of VPD using a generalized linear model with a binomial error distribution. The parameters of the model allowed calculating the maximum stomatal conductance and the VPD at which the modeled stomatal conductance was maximal.

### Trait Measurements

A total of 34 leaf and wood traits were assessed to analyze possible relationships with Ψ_50_ and K_S_ (see Table 2). To accomplish this, four total sets of samples were taken: 1) A set of five individuals with five leaves being sampled per individual for the traditional leaf traits, such as absolute area per leaf, leaf fresh-weight, leaf dry-weight, leaf nitrogen concentration (LNC) and leaf carbon concentration (LCC). The data were used to calculate specific leaf area (SLA), leaf dry matter content (LDMC) and carbon to nitrogen ratio (CN). We also determined leaf habit (deciduous/evergreen), leaf pinnation (pinnate or simple), leaf margin (entire or serrate) and recorded the presence or absence of extrafloral nectaries. 2) Another 30 leaves were sampled from three individuals per species to determine leaf tensile strength as a measure of leaf toughness. Leaf tensile strength was measured with a tearing apparatus modified after Hendry [36]. 3) The same leaves on which stomatal conductance was measured (see above), were taken to analyze the stomatal related traits. Stomatal traits were analyzed after Gerlach [37], with stomata being counted on a minimum area of 50,000 µm^2^ on three leaves from three individuals per species. Stomata were counted on nail polish impressions made on leaf samples, which had been stored in 70% ethanol. Length and width of three stomata per replicate were measured, and stomatal density was expressed as stomatal number per area. The analysis was performed with a light-optical microscope (Zeiss Axioskop 2 plus) and using the Axio Vision (Version 3.0) software. 4) A sample was taken from each twig used in the cavitation sensitivity analysis for further xylem anatomical investigation. Twig sections were prepared for light microscopic inspection and, from an area of 4.4 mm2 per sample, every xylem vessel was analyzed. To determine xylem traits, we made use of XylemDetector that was implemented as part of the open-source package MiToBo (http://www.informatik.uni-halle.de/mitobo), an extension of the Java image processing software ImageJ. We measured the mean lumen area of conducting vessels (MEANAREA) and the mean roundness of conducting vessels (MEANROUND), which is a measure of how close the vessel shape is to a perfect circle, and ranges from 0 to 1. MEANROUND was calculated as:
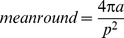
where a is the area and p the perimeter of the lumen.

**Table 2:** List of the leaf traits measured across the 39 tree species.

Code	Trait	Analytical technique	Type	Units/categories
Ψ_50_	Loss of 50% initial conductivity	Pressure Chamber	Continuous	MPa
K_S_	Maximum conductivity	Pressure Chamber	Continuous	kg m^−1^ s^−1^ MPa^−1^
b	Parameter b (Sigmoid Regression)	Pressure Chamber	Continuous	nondimensional
CONMEAN	Average stomatal condutance	Porometer	Continuous	mmol m^2^ s^−1^
CONMAX	Maximum stomatal condutance	Porometer	Continuous	mmol m^2^ s^−1^
VPDMAX	VPD at CONMAX	Porometer, Hygrometer, Thermometer	Continuous	hPa
CONMAXFIT	Fitted Max. stomatal conductance	Porometer, Hygrometer, Thermometer	Continuous	nondimensional
VPDMAXFIT	VPD at CONMAXFIT	Porometer, Hygrometer, Thermometer	Continuous	hPa
VPDPOI	VPD at point of inflection of fitted stomatal conductance	Porometer, Hygrometer, Thermometer	Continuous	hPa
SLA	Specific leaf area	Scanner, Balance	Continuous	m^2^ kg^−1^
LOG10LA	Decadic log (Leaf Area)	Scanner	Continuous	mm^2^
LDMC	Leaf dry matter content	Balance	Continuous	mg g^−1^
LT	Leaf toughness	Leaf toughness device	Continuous	N mm^−1^
LEAFHABIT	Leaf habit	Literature	Binary	(0) evergreen; (1) deciduous
LNC	Leaf nitrogen concentration	CN Analyzer	Continuous	mg g^−1^
LCC	Leaf carbon concentration	CN Analyzer	Continuous	mg g^−1^
CN	Carbon-nitrogen ratio	CN Analyzer	Continuous	ratio
CA	Leaf calcium concentration	AAS Analyzer	Continuous	mg g^−1^
K	Leaf potassium concentration	AAS Analyzer	Continuous	mg g^−1^
MG	Leaf magnesium concentration	AAS Analyzer	Continuous	mg g^−1^
LEAFPIN	Leaf pinnation	Field Observation	Binary	(0) pinnate; (1) simple
LEAFMAR	Leaf margin	Field Observation	Binary	(0) dentate; (1) entire
EXTRAFLO	Extrafloral nectaries	Field Observation	Binary	(0) no; (1) yes
STODENS	Stomata density	Microscopy	Continuous	1 mm^−2^
STOSIZE	Stomata size	Microscopy	Continuous	µm^2^
STOIND	Stomata index	Microscopy	Continuous	ratio
DIAMVEIN1	Diameter veins 1st order	Scanner	Continuous	cm
DIAMVEIN2	Diameter veins 2nd order	Scanner	Continuous	cm
VEINDENS	Length of veins per unit leaf area	Scanner	Continuous	cm cm^−2^
WPOT	Water potential	Pressure Chamber	Continuous	MPa
WOODDENS	Wood density	Balance	Continuous	g cm^3^
MEANAREA	Mean area of conducting vessels	Microscopy	Continuous	µm^2^
MEANROUND	Mean roundness of conducting vessels	Microscopy	Continuous	nondimensional
DHYD	Hydraulically weighted diameter of conducting vessels	Microscopy	Continuous	µm

All traits were assessed on the individuals planted in the experiment. The table includes the trait abbreviations (Code) used throughout the text.

Following Sperry [38], we calculated hydraulically weighted conduit diameter (DHYD) from the lumen area data according to:
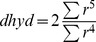
where r are the circle radii calculated from the lumen areas.

### Statistical Analyses

**Figure 3:**
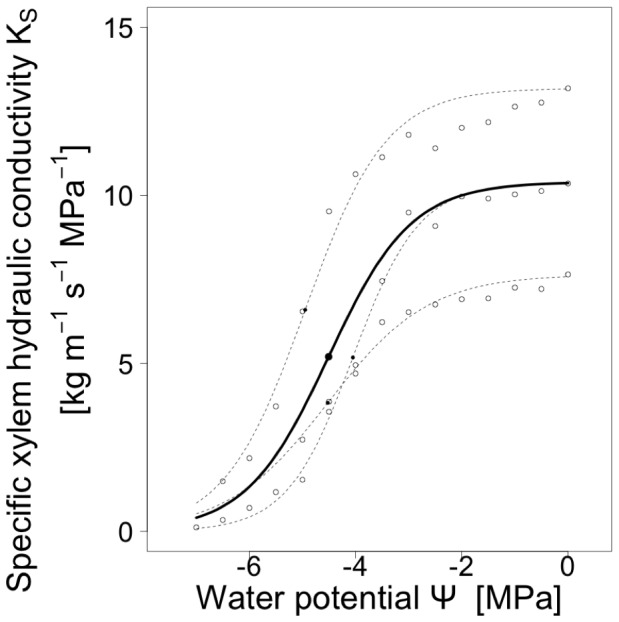
Conductivity rates as a function of decreasing water potential, taking *Castanea henryi* as an example. Outline dots show the measured values of three independent vulnerability curves. Dotted lines show the single regression functions obtained from the measured value per vulnerability curve, obtained from a 3-parametric sigmoid function. The bold line shows the regression lines constructed from the means of the parameters from the three single vulnerability curves. Filled dots represent Ψ_50_ values.

We plotted vulnerability curves that show the flow rates of perfusion solution through stem segments as a function of water potential [35]. A sigmoid, three-parameter regression was applied to the vulnerability data [39],[40]:
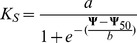
where K_S_ is the specific hydraulic conductivity of the xylem [kg m^−1^ s^−1^ MPa^−1^], Ψ is the xylem pressure at which water flow was measured [MPa], a is the original maximum specific xylem hydraulic conductivity, b is the slope of the regression and Ψ_50_ is the xylem pressure at which 50% loss of the original maximum specific xylem hydraulic conductivity occurs. Fig. 3 shows an example of how the sigmoid model was fitted to predict the loss of specific xylem hydraulic conductivity (K_S_) from water potential (please refer to the Figure S1 and Table S1 for raw data on xylem vulnerability). We made use of the FactoMineR package in R (http://cran.r-project.org/web/packages/FactoMineR/index.html) to correlate the physiological parameters to the species' traits in a PCA. K_S_, Ψ_50_ and the slope b of the K_S_ ∼ Ψ relationship were tested for differences between deciduous and evergreen leaf habit by analysis of variance (ANOVA) and for bivariate relationships to leaf traits by linear regression models. As these tests performed multiple testing, they run the risk of error inflation and cannot be used to infer statistical significances. These tests were exploratory and had the purpose to identify possible candidate predictors and to show the direction of their effects. To further investigate the emerging significant relationships, we rerun all significant linear regressions by additionally including the interaction with leaf habit. For all statistics, R software version 3.0.2 was used.

## Results

**Figure 4:**
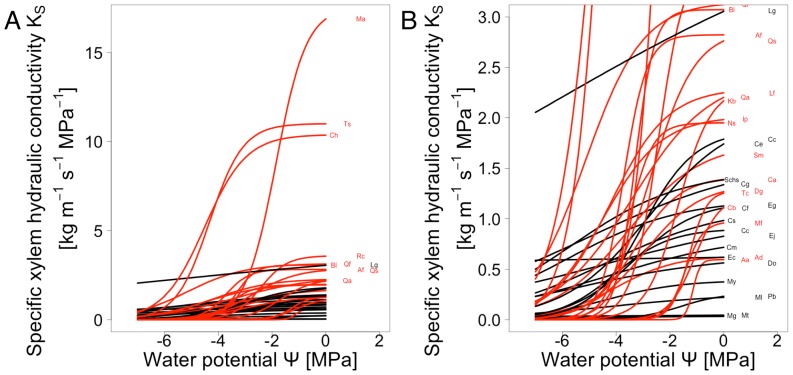
Plots of modeled specific xylem hydraulic conductivity K_S_ versus water potentials for all 39 species included in the study. A) and B) show the same data at different scale of K_S_. Deciduous species are shown in red, evergreen species shown in black. For species abbreviations see Table 1. For details of calculation of regression lines, see Fig. 2 and Methods.

**Figure 5:**
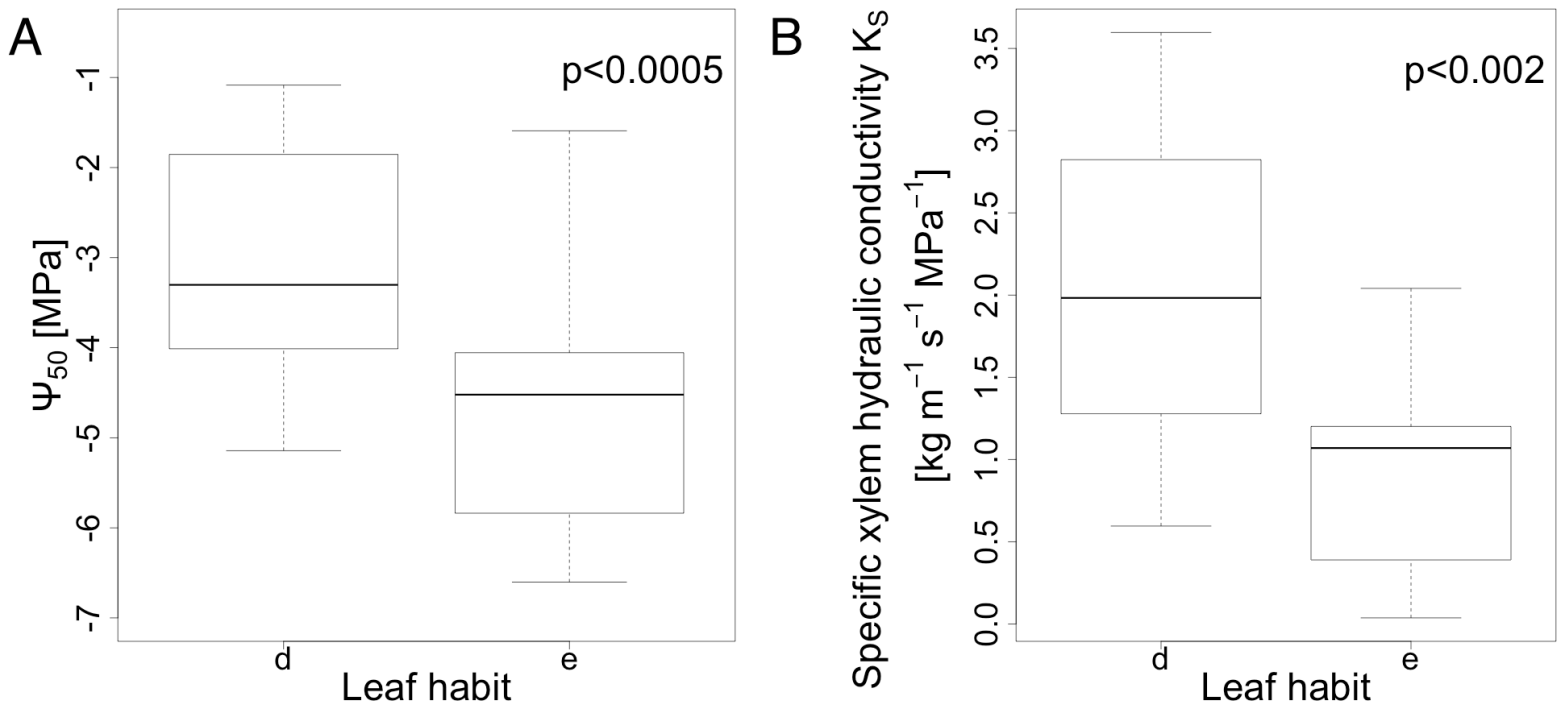
Boxplots characterizing the species set by leaf habit. A) xylem pressure at 50% loss of the maximum specific xylem hydraulic conductivity (Ψ_50_) and B) specific xylem hydraulic conductivity of the xylem (K_S_) as a function of leaf habit. d  =  deciduous, e  =  evergreen. The probability values were derived from an ANOVA.

Fig. 4 shows the vulnerability curves for all 39 species included in the study. Values of Ψ_50_ ranged between −1.08 MPa and −6.6 MPa for *Celtis biondii* and *Lithocarpus glaber*, respectively, with an overall mean of −3.78 MPa (SD = 1.48). Specific xylem hydraulic conductivity (K_S_) was highest in *Melia azedarach, Triadica sebifera* and *Castanea henryi* (17.52, 11.01 and 10.40 kg m^−1^ s^−1^ MPa^−1^, respectively) and lowest in *Machilus grijsii* (0.036 kg m^−1^ s^−1^ MPa^−1^), with a overall mean of 2.44 kg m^−1^ s^−1^ MPa^−1^ (SD = 3.31). K_S_ and Ψ_50_ were not correlated across all species (p = 0.512). Evergreen species had significantly lower values of maximum hydraulic conductivity and lower Ψ_50_ values than deciduous species (Fig. 5).

**Figure 6:**
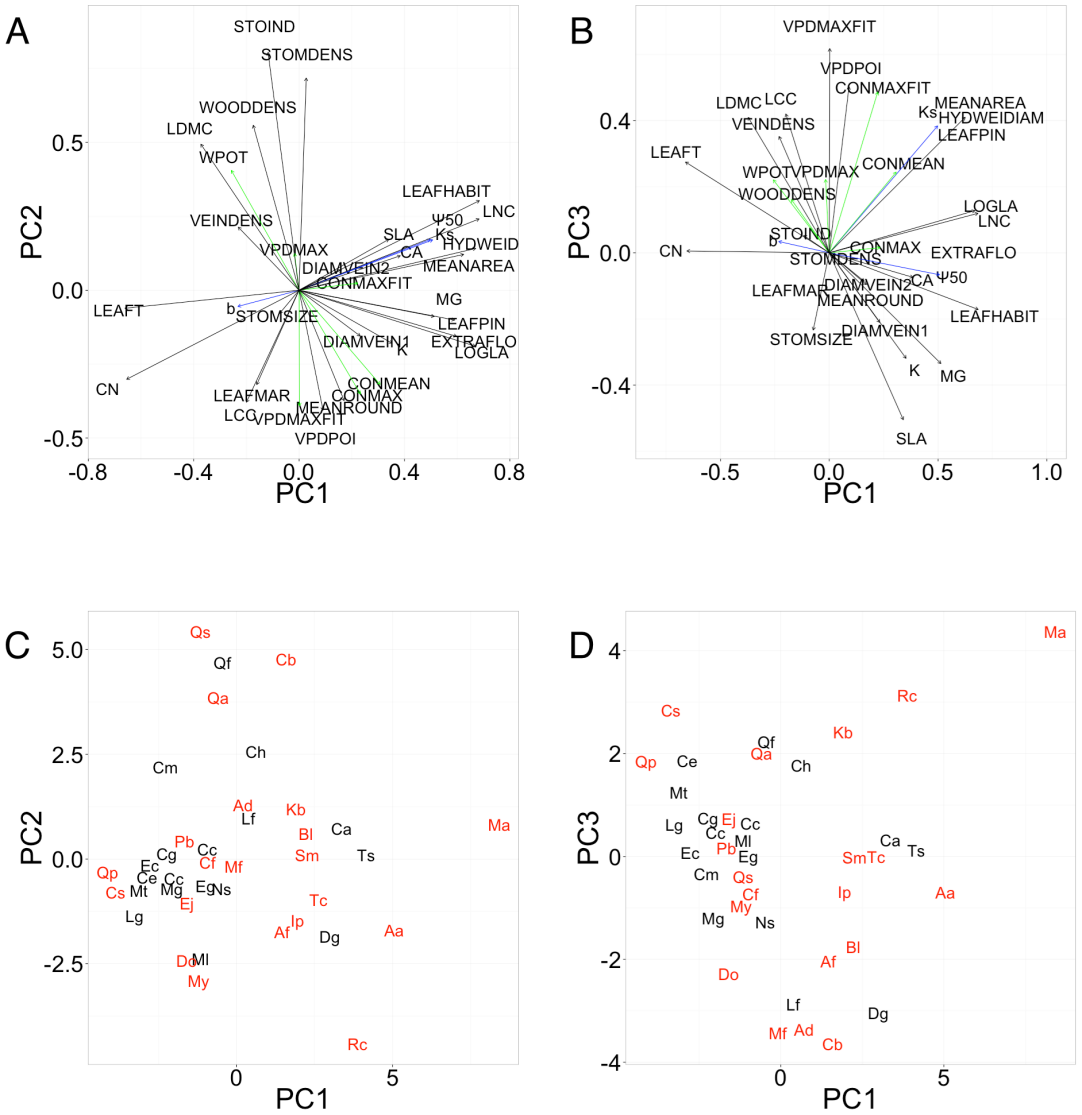
Principal component analysis (PCA) biplots showing the relationships between the mean values of all chemical and morphological leaf traits (black arrows), all parameters of stomatal regulation (green arrows) and all parameters of cavitation sensitivity (blue arrows). A) and C) PCA axes 1 and 2, B) and D) PCA axes 1 and 3. A) and B) loadings of the different traits. C) and D) species scores in the PCA shown separately by leaf habit. Deciduous species are shown in red and evergreen species in black. See Table 1 for abbreviations of species names. Eigenvalues: axis 1 = 7.03, axis 2 = 4.2, axis 3 = 3.48, with cumulative proportion of explained inertia 20.1%, 33.0% and 43.3%, respectively. See Table 2 for abbreviations of trait names.

Fig. 6 shows the principal components analysis (PCA) of all 34 leaf traits, including the parameters of stomatal control and xylem vulnerability for all 39 study species (Fig. 6 a, b). The species mean values of all traits are provided in Table S1. The first three PCA axes explained 43.3% of the total variance, with eigenvalues of 7.03, 4.2 and 3.48, respectively. While evergreen species tended to score higher on the first PCA axes than deciduous species, there was a large overlap between the two leaf habits (Fig.6 c, d). Positive scores on the first PCA axis reflected both decreasing xylem vulnerability and increasing values of traits of the leaf economics spectrum, such as leaf nitrogen concentration (LNC) and specific leaf area (SLA), as well as evergreen leaf habit and the logarithm of the area of a single leaf (Log10LA), while leaf toughness (LEAFT) and leaf carbon to nitrogen ratio (CN) showed negative loadings. Parameters of stomatal control were correlated with the second PCA axis, with positive loadings being recorded for stomatal index (STOIND), stomatal density (STOMDENS) and wood density (WOODDENS), and negative ones for the point of inflection of the g_s_ ∼ VPD curve (VPDPOI) and maximum stomatal conductance (CONMAX).

**Figure 7:**
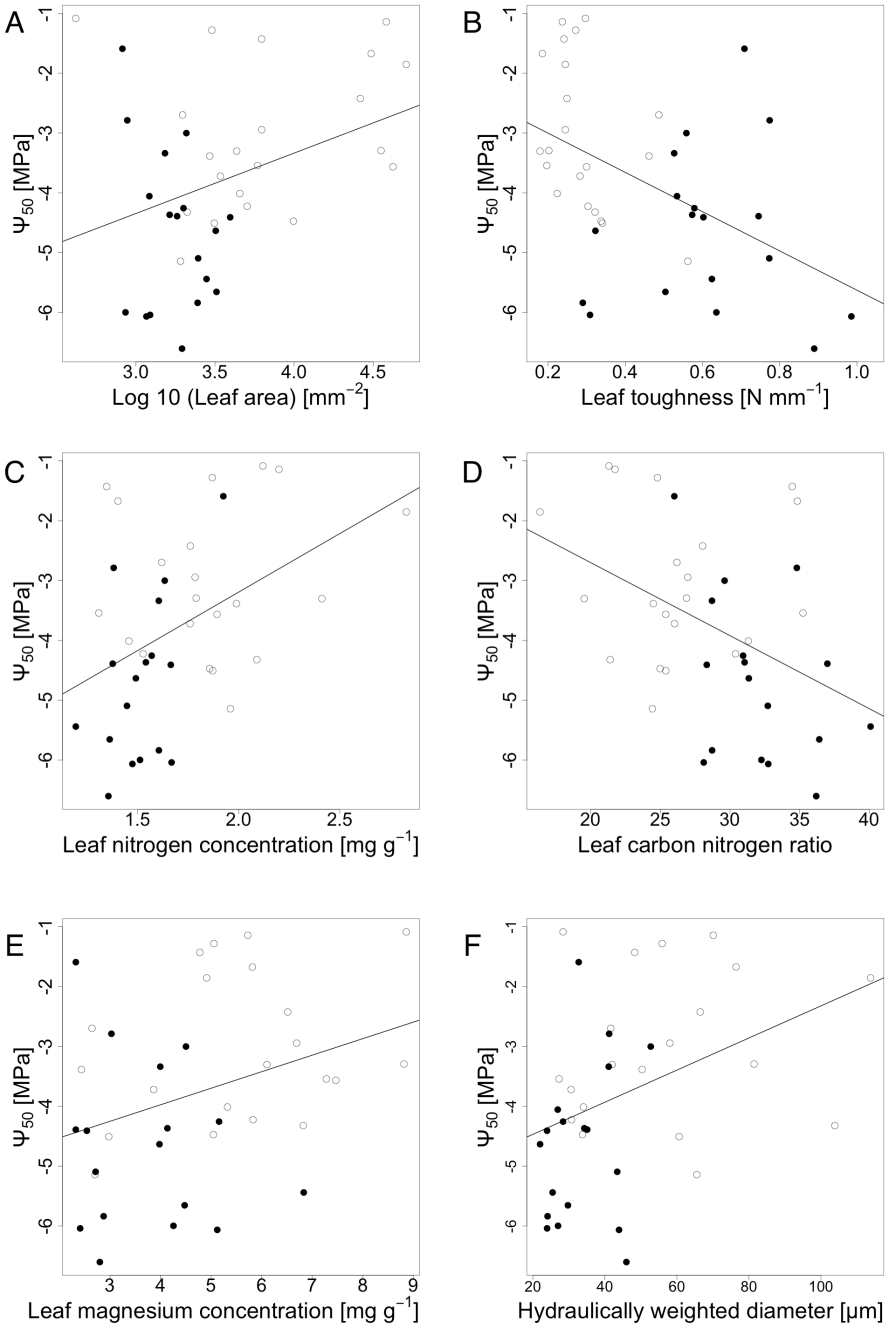
Ψ_50_ as a function of A) leaf area (p = 0.0315, r = 0.34), B) leaf toughness (p<0.0003, r = −0.47), C) leaf nitrogen concentration (p<0.0075, r = 0.43), D) leaf carbon to nitrogen ratio (p<0.0078, r = −0.43), E) leaf magnesium concentration (p<0.042, r = 0.33) and F) hydraulically weighted conduit diameter (p<0.01, r = 0.39). Filled black dots represent species of evergreen leaf habit; empty dots represent species of deciduous leaf habit.

**Figure 8:**
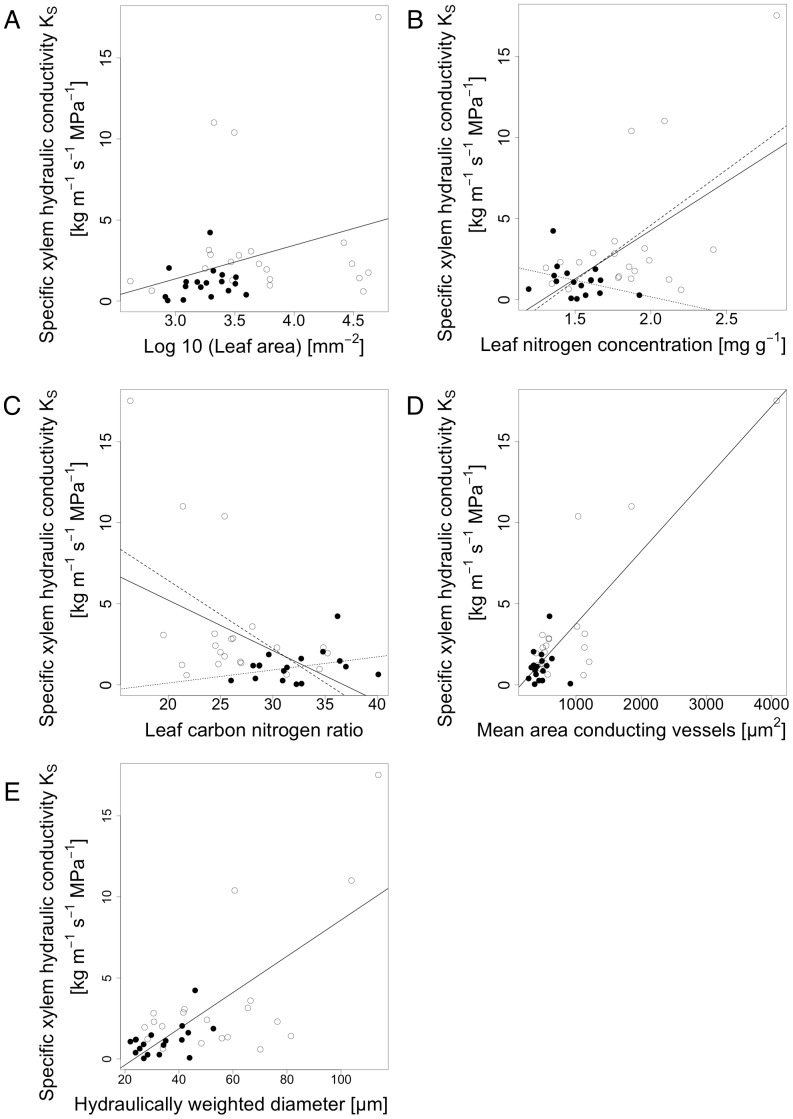
K_S_ as a function of A) leaf area (p<0.044, r = 0.1), B) leaf nitrogen concentration (p<0.00001, r = 0.59), C) leaf carbon to nitrogen ratio (p<0.0019, r = 0.48), D) mean area of conducting vessels (p<0.0001, r = 0.85), and E) hydraulically weighted diameter of conducting vessels (p<0.0001, r = 0.72). Filled black dots represent species of evergreen leaf habit and empty dots represent species of deciduous leaf habit. In B) and C) K_S_ also showed significant interactions with leaf habits. Dotted lines represent species of evergreen and broken lines represent species of deciduous leaf habit. B) leaf habit p = 0.061, interaction leaf nitrogen concentration and leaf habit p = 0.049, C) leaf habit p = 0.026, interaction leaf carbon to nitrogen ratio and leaf habit p = 0.031.

We found xylem vulnerability to be significantly related to numerous traits (Table 3, Fig. 7 & 8). Ψ_50_ was positively correlated with leaf area, LNC, hydraulically weighted diameter and leaf magnesium concentration, while it was only marginally significantly related to leaf calcium concentration, and negatively related to leaf toughness and carbon to nitrogen ratio. A similar pattern was found for maximum hydraulic conductivity (K_S_), which showed a positive relationship to leaf area (Log10LA), leaf nitrogen concentration (LNC) and two morphological wood traits (i.e. the mean area of conducting vessels (MEANAREA), and the hydraulically weighted diameter) and a negative correlation with leaf carbon to nitrogen ratio (CN). The regression parameter b was not related to any of the traits studied. The regression equations from the significant linear models are shown in Table 4. There were no significant correlations between Ψ_50_ or K_S_ to any parameter of stomatal control.

**Table 3:** Correlation matrix of hydraulic xylem characteristics with numerical leaf traits and parameters of stomatal control.

	Ψ_50_		K_S_		b	
	r	p	r	p	r	p
CONMEAN	0.12	0.49	0.13	0.42	−0.12	0.49
CONMAX	0.02	0.92	0.02	0.91	−0.06	0.73
VPDMAX	−0.04	0.81	0.15	0.36	0.14	0.39
CONMAXFIT	0.14	0.41	0.26	0.12	−0.11	0.50
VPDMAXFIT	−0.13	0.43	0.18	0.26	−0.01	0.96
VPDPOI	0.13	0.44	0.13	0.44	−0.10	0.53
SLA	0.27	0.09	0.05	0.78	−0.17	0.30
LOG10LA	**0.34**	**0.03**	**0.10**	**0.04**	−0.20	0.22
LDMC	−0.02	0.91	−0.02	0.91	−0.03	0.85
LEAFT	−**0.47**	**0.00**	−0.23	0.16	0.02	0.90
LNC	**0.43**	**0.01**	**0.59**	**0.00**	−0.08	0.62
LCC	−0.23	0.17	−0.12	0.49	0.05	0.76
CN	−**0.43**	**0.01**	−**0.48**	**0.00**	0.05	0.75
CA	0.32	0.05	0.09	0.58	−0.16	0.35
K	0.16	0.33	0.05	0.75	−0.13	0.44
MG	**0.33**	**0.04**	0.02	0.91	−0.24	0.15
STOMDENS	0.09	0.58	0.07	0.65	−0.08	0.64
STOMSIZE	0.06	0.74	−0.06	0.71	−0.01	0.96
STOIND	0.11	0.52	0.01	0.69	−0.09	0.59
DIAMVEIN1	0.20	0.23	−0.09	0.59	−0.23	0.17
DIAMVEIN2	0.17	0.33	0.01	0.93	−0.14	0.42
VEINLENGTH	−0.15	0.37	−0.09	0.61	0.19	0.24
WPOT	0.09	0.57	−0.04	0.79	0.01	0.95
WOODDENS	−0.06	0.73	0.06	0.71	−0.13	0.42
MEANAREA	0.24	0.15	**0.85**	**0.00**	−0.11	0.52
MEANROUND	0.04	0.82	−0.01	0.94	−0.02	0.92
DHYD	**0.39**	**0.01**	**0.72**	**0.00**	−0.20	0.23

The correlations were calculated from linear model analyses between functional leaf traits and the extracted physiological parameters. Significant relationships are shown in bold letters. Ψ_50_ =  xylem pressure at which 50% loss of the original maximum specific xylem hydraulic conductivity occurred, K_S_ =  specific hydraulic conductivity of the xylem, b =  slope of the three-parametric sigmoid model of the vulnerability curve, CONMEAN  =  mean g_s_; CONMAX  =  mean g_s max_; VPDMax  =  VPD at g_s max_; CONMAXFIT  =  modeled g_s max_; VPDMaxfit  =  VPD at modeled g_s max_, and VPDPoi  =  VPD at the point of inflexion of the g_s_ ∼ VPD curve, MEANAREA  =  the mean area of conducting vessels, MEANROUND  =  the mean roundness of conducting vessels, and DHYD the hydraulically weighted diameter of conducting vessels.

**Table 4:** Regression equations for all significant correlations of Ψ_50_ and K_S_ to the functional traits.

Xylem parameter	Functional trait	Equation
Ψ_50_	LOG10LA	y = 1.0090x−7.3717
Ψ_50_	LEAFT	y = −3.2808x−2.3454
Ψ_50_	LNC	y = 1.9556x−7.1038
Ψ_50_	CN	y = −0.12210x−0.25799
Ψ_50_	MG	y = 0.2763x−5.0792
Ψ_50_	DHYD	y = 0.0136x−5.005
K_S_	LOG10LA	y = 2.0699x−4.8375
K_S_	LNC	y = 6.003x−7.734
K_S_	CN	y = −0.31132x+11.44974
K_S_	MEANAREA	y = 0.0011481x−0.7089940
K_S_	DHYD	y = 0.0569x−2.611

The correlations were calculated from linear model analyses. Ψ_50_ =  xylem pressure at which 50% loss of the original maximum specific xylem hydraulic conductivity occurred, K_S_ =  specific hydraulic conductivity of the xylem, b =  slope of the three-parametric sigmoid model of the vulnerability curve, CONMEAN  =  mean g_s_; CONMAX  =  mean g_s max_; VPDMax  =  VPD at g_s max_; CONMAXFIT  =  modeled g_s max_; VPDMaxfit  =  VPD at modeled g_s max_, and VPDPoi  =  VPD at the point of inflexion of the g_s_ ∼ VPD curve, MEANAREA  =  the mean area of conducting vessels, and DHYD the hydraulically weighted diameter of conducting vessels.

Including leaf habit in the significant models resulted in two models with significant interactions with leaf habit, i.e. for leaf nitrogen concentration (LNC) and the leaf carbon to nitrogen ratio (CN). While with increasing CN and decreasing LNC, K_S_ increased in evergreen species, deciduous species showed a decrease (Fig. 8 a, b).

## Discussion

We found a large variation in specific xylem hydraulic conductivity and xylem vulnerability among our study species, which points to different complementary strategies of the species in the same subtropical forest community. Thus, our results conform to the findings of Böhnke *et al.* [41], who revealed a high and temporally constant level of functional diversity in the course of succession in these forests. In particular, variation in cavitation resistance could offer an explanation for species coexistence in the same community [42]. Our values on specific xylem hydraulic conductivity with a range of K_S_ between 0.036 and 17.52 kg m^−1^ s^−1^ MPa^−1^ and xylem vulnerability to cavitation Ψ_50_ between −1.08 and −6.6 MPa cover a large part of the total range recorded for such variables in other studies [43]. For example, Cavender-Bares *et al.* [44] described a range of maximum specific xylem hydraulic conductivity of between 1.75 and 5 kg m^−1^ s^−1^ MPa^−1^ for 17 oak species, while Maherali *et al.* [45] reported a mean of 1.36 kg m^−1^ s^−1^ MPa^−1^ of maximum specific xylem hydraulic conductivity (n species  = 87) and a mean of −3.15 MPa for Ψ_50_ values (n species  = 167). Slightly higher Ψ_50_ values of −1.2 up to −2.76 MPa were encountered for eight tree species from a tropical dry forest [46]. However, we have to consider that extreme values of K_S_ of more than 10 kg m^−1^ s^−1^ MPa^−1^ might be methodological artifacts, caused by some open vessels in these samples. However, the three species with extreme K_S_ also ranked high in values of the predictor traits for K_S_, indicating that the relative rank in K_S_ in these species might be realistic. As vessel length of the species was not measured, and thus, in some species, specific xylem hydraulic conductivity might have been overestimated, comparisons to other studies should be done with caution. However, as Melcher *et al*. [33] pointed out, such an overestimation is probably not severe, as long vessels are also very rare.

Important leaf traits such as leaf nitrogen concentration (LNC) and leaf area (Log10LA) were highly correlated to Ψ_50_ and K_S_. Thus, we can fully confirm our first hypothesis that leaf traits describing the leaf economics spectrum are related to specific xylem hydraulic conductivity and cavitation resistance. Interestingly, there were no significant correlations for some of the traits of the leaf economics spectrum, such as SLA and LDMC. Thus, leaf thickness and water content of leaves seem to have less importance for hydraulic characteristics than the leaves' absolute size and protein content. This contrasts with the findings reported by Willson *et al.* [47], who described a significant relationship between SLA and Ψ_50_ for the genus *Juniperus*. Thus, comparative studies confined to certain taxonomic levels, such as congeneric comparisons, might arrive at different conclusions than studies covering a wider range of taxa. Alternatively, SLA may have a differing level of importance with regard to the physiology of gymnosperms and angiosperms. Interestingly, our study did also not support a link between K_S_ and leaf vein density, which was predicted by the flux trait network suggested by Sack et al. [15]. However, there was only equivocal evidence for a significant relationship between K_S_ and leaf vein density in their reviewed studies [15].

Our results of a strong relationship of Ψ_50_ and K_S_ to leaf area, conform to those encountered for eight southern African tree species of a seasonally dry tropical forest by Vinya *et al.* [48], except that they reported a link to leaf area only for K_S_, but not for Ψ_50_. Our findings of a relationship of Ψ_50_ to Mg concentration and a marginal one to Ca concentration might indicate that the non-vein and non-sclerenchyma mesophyll density in the leaf are more relevant for cavitation resistance than overall leaf tissue density. As a central component of chlorophyll, Mg concentration is directly related to photosynthetic capacity, and thus, might capture this proportion of actively assimilating tissue in the leaf. In addition, as a cofactor of many enzymatic processes, Mg can be considered an indicator for the plant's nutrition status [49].

The absence of any direct relationship between SLA or LDMC with Ψ_50_ or K_S_ which is in accordance with Sack *et al*. [50], in combination with the large overlap in Ψ_50_ and K_S_ detected between deciduous and evergreen species, is not a conclusive result. It appears that evergreen and deciduous subtropical forest species form two ends of a gradient from cavitation resistance to cavitation avoidance, respectively. This view is supported by a recent study by Fu *et al.* [17], who investigated the relationship of stem hydraulics and leaf phenology in Asian tropical dry forest species. In particular, they found a negative relationship between leaf life span and K_S_ but no significant relationship between leaf life span and Ψ_50_. In accordance with our results, Maherali *et al*., Choat *et al*. and Chen *et al*. [27],[45],[51] also reported significant differences in Ψ_50_ and K_S_ between the different leaf habit groups. Such differences in hydraulic characteristics also translate to higher growth rates, as shown by Fan *et al*. [52] for 40 Asian tropical trees. However, some other studies failed to detect any differences, such as that of Markesteijn *et al*. [42], who attributed the substantial differences they encountered in Ψ_50_ and K_S_ to shade tolerance. They also argued that the distinction between pioneer vs. shade-tolerant species predicts hydraulic properties better than leaf habit, because there are considerable overlaps in strategies along the gradient of leaf longevity. As Givnish [53] pointed out, evergreen leaves can be advantageous under a wide range of ecological conditions, and the relationship of leaf habit with Ψ_50_ and K_S_ may therefore strongly depend on the system considered. We can also confirm our second hypothesis that evergreen species characterized by low SLA and high LDMC show lower Ψ_50_ and K_S_ values than deciduous species. Interestingly, we found leaf habit to significantly influence the relationships of K_S_ to leaf nitrogen concentration and carbon to nitrogen ratio, which might be explained by differences in basic leaf constructing principles. Deciduous species tend to invest high amounts of nitrogen to maximize photosynthetic assimilation per leaf mass, whereas in evergreen species, the focus is on increased leaf lifespan, which is reflected in higher leaf carbon concentration [54]. Furthermore, deciduous species show a more conservative stomatal control to avoid embolism, whereas evergreen species tend to have more cavitation-resistant vessels [7,25] [7],[25]–[27],[55]. The underlying reason is that evergreen species are mostly diffuse-porous, which also explains the strong impact of the hydraulically weighted conduit diameter and mean area of conducting vessels on K_S_, which is also well-known from the literature [19].

Contrary to expectations, this study did not find a significant link between xylem hydraulic conductivity and parameters of stomatal regulation. Neither the maximum stomatal conductance, the vapor pressure deficit at maximum stomatal conductance nor the vapor pressure deficit at which stomatal conductance is down-regulated was related to any parameter of the xylem vulnerability curves. As such, our third hypothesis has to be rejected, which implies that the ability of a very precise and fast stomatal regulation versus a retarded and inert stomatal regulation does not translate into cavitation resistance. Additional insights into the relationship between K_S_ and leaf stomatal regulation might be gained by calculating leaf-specific xylem hydraulic conductivity K_L_, which would directly refer to the capacity of the vascular system of a stem to supply the water to that stem [56]. However, our results confirm those of Brodribb *et al.* [46], who found no correlation between Ψ_50_ and the leaf water potential at stomatal closure in eight tropical, dry forest trees. They concluded that xylem cavitation and stomatal closure are linked through complex indirect regulatory mechanisms and argue that this potential linkage is considerably flexible, especially with regard to different leaf phenology strategies, and that there may be carry-over effects of preceding embolism events on stomatal control. A further explanation of a lacking relationship between xylem vulnerability and stomatal regulation may be the different scale at which stomatal regulation is considered. At the level of whole trees, Litvak *et al*. [55] found a strong linear relationship between the sensitivity of tree-level sap flow to VPD and Ψ_50_ both within diffuse- and ring-porous species, which was not encountered for leaf-level transpiration rates. The authors argue that the tree-level transpiration sensitivity, in addition to stomatal regulation, also directly responds to drought-induced embolisms.

Several studies showed a trade-off between high hydraulic conductivity and cavitation resistance [57]–[60]. In contrast to these studies, we found Ψ_50_ and K_S_ to be unrelated. According to our current understanding of the causes of xylem embolism under drought conditions, there may indeed be no mechanistic link between these two hydraulic characteristics. As such, diameter and length of vessels may differ autonomously from pit structure and size [19]. While K_S_ is mainly driven by vessel diameter, Ψ_50_ depends on pit size and structure [19],[20]. The pit area hypothesis states that cavitation resistance is linked to the total area of inter-vessel pits per vessel [61],[62]. Thus, the risk of an embolism expanding between vessels rises with the maximum size of the pit membrane pore, which in turn is dependent on the associated pit membrane area per vessel. This was demonstrated by Hacke *et al.* [57], who reported a strong negative link between xylem vulnerability and pit membrane area per vessel, resulting in small pits potentially increasing hydraulic resistance and decreasing K_S_. However, pit size may be of minor importance to K_S_ compared to that of vessel diameter, and the relationship of hydraulic conductivity and cavitation resistance might depend on the specific ecosystem considered. Tyree *et al.* [59] distinguished between frost- and drought-induced cavitation. In their meta-analysis, the trade-off between hydraulic conductivity and cavitation resistance was mainly related to frost-induced cavitation events. Although frosts occur in the Chinese subtropics, they are neither very strong, nor long-lasting [63]. Thus, cavitation in the forests of our study area will mainly be brought about by drought events, which may result in far fewer, or insignificant, trade-offs.

Since all our individuals have the same age, our species set provides a high comparability usually not found in comparative studies. We expect that some of our response variables will change with tree age, such as specific xylem hydraulic conductivity [64],[65]. In addition, future comparisons should take the sustained leaf area into account, as whole-tree leaf-specific hydraulic conductance (K_L_) is known to decrease with tree age [66],[67].

## Conclusion

For the studied subtropical forest community, we demonstrated a clear link of K_S_ and Ψ_50_ with functional traits, and particularly with leaf nitrogen concentration, log leaf area and leaf carbon to nitrogen ratio. Thus, easily measured leaf traits from the LES have the potential to predict plant species' drought resistance. However, current knowledge on xylem vulnerability and traits from other ecosystems do not allow generalizing from these results. In addition, our finding of an absence of any relationship between parameters of stomatal control raises the question whether stomatal control as characteristics that are an independent axis of the LES might be related to an axis of xylem characteristics that are independent of specific xylem hydraulic conductivity and xylem vulnerability.

## Supporting Information

Figure S1 **Raw data for the vulnerability curves of the 39 study species analyzed.** Filled dots represent measured data, empty dots show estimated Ψ_50_ values and the broken lines represent the fitted models of xylem vulnerability. For species abbreviations see Table 1.
(PNG)

Table S1 **Trait raw data for the 39 study species analyzed.** For trait codes and full species names, please see Tables 1 and 2.
(TXT)
