# Supplementary figures and images for: Linking Xylem Hydraulic Conductivity and Vulnerability to the Leaf Economics Spectrum—A Cross-Species Study of 39 Evergreen and Deciduous Broadleaved Subtropical Tree Species
Source: PLoS One. 2014 Nov 25;9(11):e109211. doi: 10.1371/journal.pone.0109211 (PMC4244042; doi:10.1371/journal.pone.0109211)

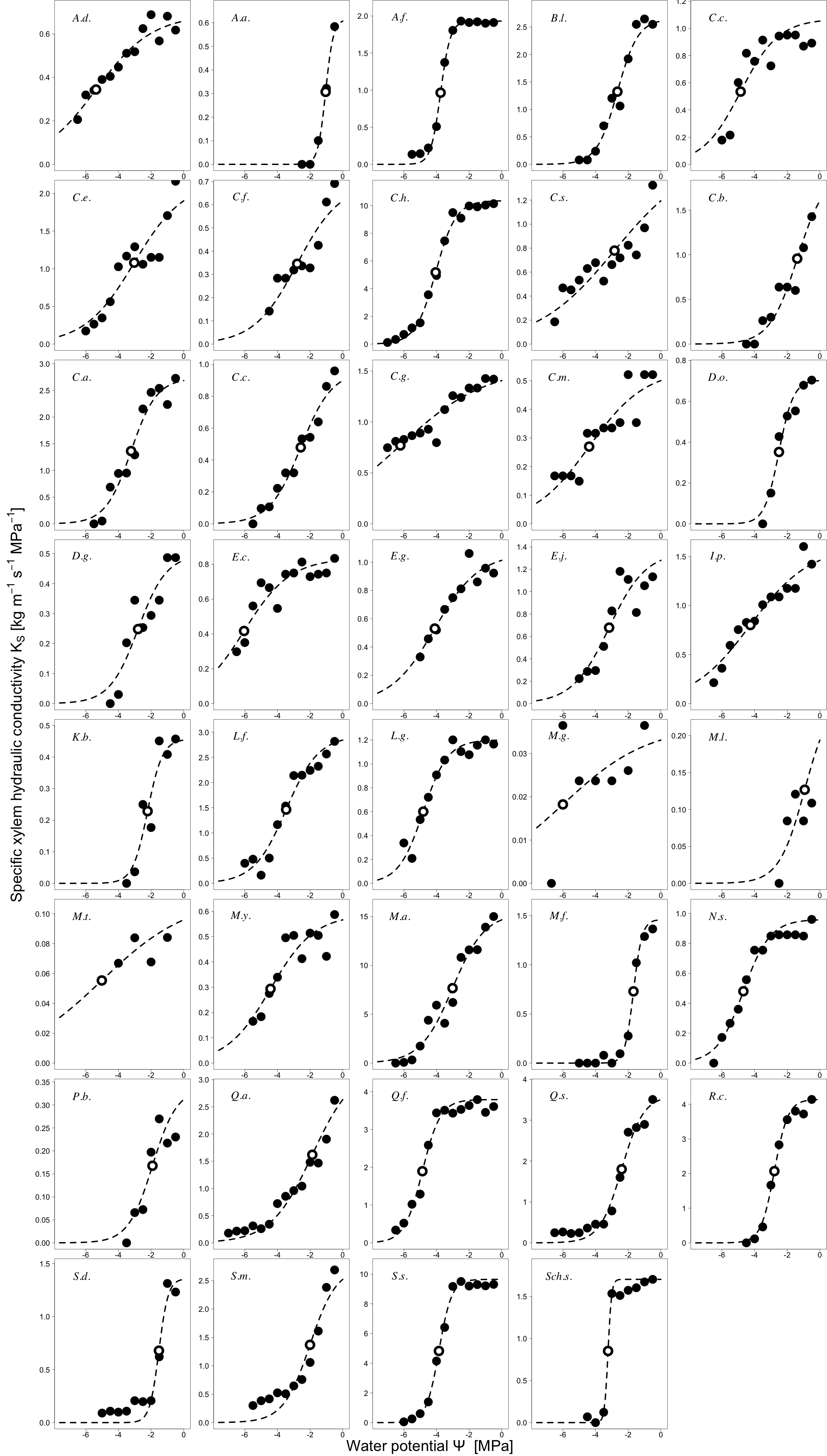

Supplement: Figure S1 — Raw data for the vulnerability curves of the 39 study species analyzed. Filled dots represent measured data, empty dots show estimated Ψ50 values and the broken lines represent the fitted models of xylem vulnerability. For species abbreviations see Table 1. (PNG) [file pone.0109211.s001.png]
